# Establishment of a Regional Interdisciplinary Medical System for Managing Patients with Tuberous Sclerosis Complex (TSC)

**DOI:** 10.1038/s41598-018-35168-y

**Published:** 2018-11-13

**Authors:** Ayataka Fujimoto, Tohru Okanishi, Shin Imai, Masaaki Ogai, Akiko Fukunaga, Hidenori Nakamura, Keishiro Sato, Akira Obana, Takayuki Masui, Yoshifumi Arai, Hideo Enoki

**Affiliations:** Facility, Tuberous Sclerosis Complex Board, Seirei Hamamatsu General Hospital, 2-12-12 Sumiyoshi, Nakaku, Hamamatsu, Shizuoka 430-8558 Japan

## Abstract

Tuberous sclerosis complex (TSC) is an autosomal dominant inherited disease characterized by lesions that involve multiple organs. Interdisciplinary management at individual facilities needs to be coordinated to treat multiple organ systems. We hypothesized that the number of patients, opportunities for patients to undergo examinations, and opportunities for patients to be treated would increase after establishment of a TSC board (TB) in our hospital. From August 1979 to August 2017, 76 patients were studied. We established the TB in our hospital in 2014. We divided the patients into the pre-TB group and post-TB group. Patients consisted of 33 females and 43 males (mean age, 18.7 years; median age, 15 years). The follow-up period was 2 to 457 months (mean, 51.6 months; median, 24.5 months). Twenty-four patients were in the pre-TB group, and 52 were in the post-TB group. Regular follow-up (p < 0.001), younger age (p = 0.002), opportunities for patients to undergo examinations, opportunities for patients to receive neurological treatment (p < 0.001), and mammalian target of rapamycin (mTOR) inhibitor usage (p = 0.041) were significantly higher in the post-TB group. The radial relationship around the axis of TSC coordinators may be the key to interdisciplinary management of TSC.

## Introduction

Tuberous sclerosis complex (TSC) is an autosomal dominant inherited disease characterized by lesions that involve multiple organs of the body and variable clinical manifestations^1^. Although TSC shows dominant inheritance, 60–70% of patients are sporadic cases due to de novo mutation. The incidence rates for TSC range between 1/6000 and 1/10000 live births, and the prevalence rates for TSC were reported as 1/7000 to 1/20000^2–7^.

*TSC1* is located at 9p34 and encodes the hamartin protein. *TSC2* is located at 16p13.3 and encodes the tuberin protein. The functional complex of hamartin and tuberin acts as a GTPase that activates Ras homolog enriched in brain (Rheb) protein. Rheb-GTP activates mammalian target of rapamycin (mTOR), but the hamartin-tuberin complex suppresses mTOR activity by converting Rheb-GTP to Rheb-GDP^8^. Mutations in either *TSC1* or *TSC2* result in constitutive upregulation of the mTOR pathway, leading to hamartoma formation owing to the reduced function of this complex due to the gene mutation^9^. Thus, TSC can affect virtually every organ, with brain, kidneys, lungs, heart, and skin most frequently involved.

The various symptoms of TSC are age dependent^10^. Cardiac rhabdomyoma occurs in the fetal period and mostly disappears in infancy. Hypomelanotic macules and cortical/subcortical tubers that were present since infancy do not increase in number or size. Subependymal giant cell astrocytomas (SEGAs) mainly occur from infancy to adolescence. Facial angiofibroma (AF) and renal angiomyolipoma (AML) mainly occur after school age and increase. Thus, patients with TSC require medical treatment throughout their life from a well-organized team that integrates approaches to separate disciplines into a single consultation^11^.

In the United Kingdom (UK), some parts of Europe, and the United States (US)^12–14^, well-organized communities are in place that provide better treatment, care, a regional network, education about TSC, and research foundations with a long history. However, many other countries like Japan do not have such a strong community for patients with TSC.

Although TSC is familiar to pediatricians, few experts who see adult patients are familiar with systemic clinical practice. Additionally, bureaucratic relationships are often present among departments, and a TSC patient likely has to go to multiple clinics and departments in different facilities to be treated by individual experts over the years.

Treatment for TSC has recently received attention due to the introduction of mTOR inhibitors^15–17^ for the treatment of TSC-related angiofibroma^15^, SEGAs^18^, AML^19,20^, and epilepsy^21^, further complicating the medical system. mTOR inhibitors have systemic effects on patients, but specialists may only pay attention to the organ(s) in which they specialize without considering other positive and negative systemic effects. Although we know that in Japan, we cannot immediately introduce the types of systems present in countries such as the UK and US, introducing a similar system could allow more efficient TSC practice. Therefore, we established a compact system that can comprehensively and cross-sectionally treat patients with TSC in our hospital.

We hypothesized that the number of patients, opportunities for patients to undergo examinations, and opportunities for patients to be treated would increase after establishment of the TSC board (TB) in our hospital. The purpose of this study was:

1. to present the process of introducing a TB in our facility,
2. to review clinical manifestations, and
3. to assess whether these patients with TSC underwent appropriate examinations and treatments.

## Results

### Patients

All the clinical data are shown in Table 1.

**Table 1:** Clinical information and survey rate of each examination.

Age (years)	27.0 (3–63)	14.8 (1–70)	0.002^†^
Gender	F:M 11:13	F:M 23:29	n.s.^††^
Follow-up period, months (range)	107.7 (4–457)	17.0 (2–33)	<0.001^†^
Intellectual functions			n.s.^‡^
Normal	12	25	
Moderate impairment	4	4	
Severe impairment	4	23	
n/a	4	0	
Loss to follow-up	10 (41.7%)	0 (0%)	n/a
Survey of each examination (per month) [mean (range)]			
Dermatological visual inspection	0.0036 (0–0.02)	0.074 (0–0.5)	<0.001^†^
Brain CT/MRI/ultrasonography	0.018 (0–0.25)	0.091 (0–0.5)	0.0104^†^
Renal MRI/CT/ultrasonography	0.005 (0–0.02)	0.079 (0–0.5)	<0.001^†^
Ultrasonography/CT	0.003 (0.−0.02)	0.068 (0–0.5)	0.014^†^
Lung CT	0.003 (0–0.02)	0.025 (0–0.5)	0.001^†^
Dental visual inspection	0 (0)	0.067 (0–0.5)	<0.001^†^
Genetic test	0.00008(0–0.02)	0.027(0–0.5)	<0.001^†^
Fundus examination	0.001 (0–0.02)	0.029 (0–0.33)	0.0035^†^

TSC: tuberous sclerosis complex; n.s.: not statistically significant; CT: computed tomography; MRI: magnetic resonance image; IF: intellectual function; Normal level: ≥70 full intellectual quotient (IQ) of Wechsler Adult Intelligence Scale III, Wechsler Intelligence Scale for Children, developmental quotient (DQ), or patients who were verbal and independent in their lives,Moderate impai rment level: full IQ ≥ 50 and < 70, DQ ≥ 30 and < 70, or patients who were verbal but dependent on others in their lives; Severe impairment level: < 50 in IQ, < 30 in DQ, or non-verbal and dependent on others in their lives. ^†^Welch t-test, ^††^Fisher exact test, ^‡^Mann-Whitney U-test; n/a: not applicable.

Numbers of patients, sex, age, and follow-up period of the pre- and post-tuberous sclerosis complex board (TB) groups are shown. This table also shows a comparison of the survey rate for each specialty between pre- and post-TB patients. The numbers in the survey of each examination were obtained by dividing the number of inspections by the follow-up period. That is, they show how many examinations were performed per month.

### TSC board

Ten patients (41.7%) in the pre-TB group discontinued visiting our hospital and were not available for follow-up (No. 4, 5, 8, 9, 10, 11, 13, 21, 22, and 23). In the post-TB group, no patients discontinued visiting our hospital. The follow-up rate was significantly different (p < 0.001) between the pre-TB and post-TB groups (Table 1).

The age of patients in the pre-TB group ranged from 3 to 63 years (mean, 27.0 years; median, 26 years), whereas the age range of patients in the post-TB group was 1 to 70 years (mean, 14.8 years; median, 12 years). The difference in age between the pre- and post-TB groups was statistically significant (p = 0.002), with the post-TB group being younger.

We found no significant difference between the pre- and post-TB groups in clinical manifestations except for AF (Table 2).

**Table 2:** Survey rates of each clinical manifestation.

	pre-TSC board (N = 24)		post-TSC board (N = 52)		*p*-value
Age [years; mean (range)]	27.0 (3–63)		14.8 (1–70)		
Gender (F:M)	11:13		23:29		
Loss to follow-up	10 (42%)		0 (0%)		<0.001^†^
**Dermatological**					
Hypomelanoic macules (%)	11 (45%)	9/11 (82%)	47 (90%)	42/47 (89%)	n.s.
Facial angiofibroma (%)	8 (33%)	8/8 (100%)	50 (96%)	29/50 (58%)	0.041
Shagreen patches (%)	4 (16%)	4/4 (100%)	36 (69%)	27/36 (75%)	n.s.
Ungual fibromas (%)	3 (13%)	1/3 (33%)	34 (65%)	12/34 (35%)	n.s.
**Neurological**					
Epilepsy (%)	23 (96%)	17/23 (74%)	52 (100%)	45/52 (87%)	n.s.
Tubers	22(92%)	19/22(86%)	50(96%)	49/50(98%)	n.s.
Subependymal giant cell astrocytoma	20 (83%)	3 /20 (15%)	50 (96%)	9/50 (18%)	n.s.
Subependymal nodules	22 (92%)	18/22 (82%)	50 (96%)	48/50 (96%)	n.s.
Hydrocephalus	2 (8%)	0/2 (0%)	50(96%)	2/50(4%)	n.s.
Psychiatric symptoms	22 (92%)	4/22 (18%)	52 (100%)	10/52 (19%)	n.s.
**Renal**					
Renal angiomyolipomas (%)	11 (46%)	5/11 (46%)	44 (85%)	22/44 (50%)	n.s.
Renal cysts (%)	10 (42%)	2/10 (20%)	34 (65%)	10/34 (29%)	n.s.
Cardiac rhabdomyomas (%)	10 (42%)	6/10 (60%)	34 (65%)	12/34 (35%)	n.s.
Lung lymphangioleiomyomatosis	6 (25%)	1/6 (17%)	19 (37%)	5/19 (26%)	n.s.
Multifocal micronodular pneumocyte hyperplasia	6 (25%)	1/6 (17%)	19 (37%)	9/19 (47%)	n.s.
**Dental**					
Dental enamel pits	0 (−)	0/0 (−)	38 (73%)	12/38 (32%)	n/a
**Ophthalmological**					
Retinal lesions	3 (13%)	0/3 (0%)	24 (46%)	13/24 (54%)	n.s.

^†^Welch’s t test; Fisher’s exact test was used in all other analyses.; n.s.: not statistically significant; n/a.: not available.

Shows a comparison of each clinical manifestation between pre- and post-tuberous sclerosis complex board (TB) patients.

### Clinical outcome

The number of times a patient visited a particular department per month in the post-TB group was significantly higher than in the pre-TB group in each department (Table 2).

### Dermatological outcomes

AF was seen in 37 (63.8%) of 58 patients who were examined. We observed a statistically significant difference between the pre- and post-TB groups regarding AF. AF was seen more frequently in the pre-TB group than the post-TB group (p = 0.041, Table 2).

Among the patients with AF, two patients (No. 1, 76) underwent an intervention. One underwent laser treatment, and the other underwent surgical treatment.

### Neurological outcomes

Sixty-two (82.7%) of 75 patients had epilepsy. Among them, all patients except for patient No. 4 were taking anti-epilepsy drugs (The current data for patient No. 4 were not available because the patient was lost to follow-up). Among them, 13 patients (21.0%) underwent open cranial epilepsy surgery (Table 3). The surgery was performed more often in the post-TB group than the pre-TB group (p < 0.001). Two patients (3.2%) underwent vagus nerve stimulation (VNS) therapy. One (1.6%) was on a ketogenic diet.

**Table 3:** Numbers of patient with each treatment and the treatment rates.

	pre-TSC board (N = 24)		post-TSC board (N = 52)		p-value
	N	Treatment rates [per month; mean (range)]	N	Treatment rates [per month; mean (range)]	
**Neurological treatment**					
Total	8	0.011 (0–0.021)	13	0.121 (0.03–0.5)	0.030
SEGA removal	2	0.008 (0.006–0.01)	3	0.056 (0.03–0.1)	
Open cranial epilepsy surgery	5	0.014 (0.01–0.21)	8	0.109 (0.03–0.33)	
VNS	0	0	2	0.265 (0.03–0.5)	
Ketogenic diet	0	0	1	0.048	
**Nephrological**					
Total	2	0.008 (0.006–0.010)	3	0.074 (0.056–0.09)	n.s.
TAE	1	0.006	2	0.067 (0.056–0.077)	
Nephrectomy	1	0.010	1	0.09	
**mTOR inhibitor**					
Total	2	0.012 (0.006–0.017)	16	0.091 (0.03–0.5)	0.011
Everolimus	2	0.012 (0.006–0.017)	16	0.091 (0.03–0.5)	
Sirolimus	0	0	0	0	

TSC: tuberous sclerosis complex; SEGA: subependymal giant cell astrocytoma; VNS: vagus nerve stimulation; TAE: transcatheter arterial embolization; mTOR: mammalian target of rapamycin; Welch’s t test was used in all analyses.; n.s.: not statistically significant.

Shows a comparison of treatments between pre- and post-tuberous sclerosis complex board (TB) patients. The numerical value is the number of treatments divided by the follow-up period. That is, the value shows how many treatments were received per month.

Twelve (17.1%) of 70 patients who underwent magnetic resonance imaging (MRI) had SEGAs. Among the 12 patients who had SEGAs, two patients (No. 30, 39) had hydrocephalus (16.7%). Five patients (41.7%) underwent SEGA removal surgery due to hydrocephalus (No. 30, 39) or tumor growth (No. 1, 7, 62).

An mTOR inhibitor was used in patient Nos. 11 and 39 (Patient No. 39 exhibited SEGA recurrence). The SEGAs were controlled by the mTOR inhibitor in these two patients.

Regarding intellectual function, 12 (60%) of 20 patients in the pre-TB group were verbal and independent in their lives with a full intelligence quotient (IQ) ≥ 70, whereas 25 (48.0%) of 52 patients in the post-TB group were verbal and independent in their lives with a full IQ ≥ 70.

Neuropsychiatric disorders were seen in 14 (18.9%) of 74 patients.

In total, neurological treatments including open surgery, VNS, and a ketogenic diet were significantly different (p < 0.001) between the pre- and post-TB groups. More intensive neurological treatments were performed in the post-TB group than the pre-TB group.

### Renal outcome

AML was seen in 27 (49%) of 55 patients who were examined. Renal cysts were seen in 12 (27.3%) of 44 patients who were examined.

Patient Nos 1, 9, 54, 72, and 75 requested surgical or catheter treatments. Patient Nos. 9 and 75 underwent nephrectomy, and patient Nos 1, 54, and 72 underwent transcatheter arterial embolization.

### Prenatal diagnosis

One patient in the pre-TB group and 13 patients in the post-TB group were diagnosed with TSC prenatally following echocardiogram investigation (p = 0.053).

## Discussion

Compared to the pre-TB group, we found that the post-TB group underwent more necessary examinations, showing a statistically significant difference. Although this outcome may be merely due to the progress of medical treatment, our data showed that all examination rates increased similarly in the post-TB group. This may be because the post-TB group underwent the examinations in a multidisciplinary setting.

Careful observation and interdisciplinary follow-up including genetic analysis are necessary in patients with TSC and their family members^10,22,23^. However, before establishment of the TB, each specialist saw patients with TSC in their own specialty area in our facility. So, once the disease was diagnosed or the symptom was resolved by the specialist, some patients were not followed up by that specialist.

Multidisciplinary and interdisciplinary medical approaches are different. The multidisciplinary team approach utilizes the skills and experience of individuals from different disciplines, with each discipline approaching the patient from its own perspective. In contrast, the interdisciplinary team approach integrates separate disciplines into a single consultation^24^. After the establishment of the TB, we followed up all patients with an interdisciplinary approach. Most patients in the post-TB group efficiently underwent necessary examinations and received appropriate treatments. Therefore, this interdisciplinary medical system for patients with TSC was introduced to a family member of each TSC patient via a website (Family Network Committee, The Japanese Society of Tuberous Sclerosis Complex). This may be why the number of patients has increased two times within a short period in our facility.

Shepherd *et al*.^25^ pointed out the lack of management in specialist care and inadequate testing in TSC. An economic burden^26^ may exist due to the broad spectrum of manifestations that develop within multiple organ systems. Many caregivers of patients with TSC have significantly lower physical- and mental health-related quality of life scores compared to healthy adult population norms^27^. Therefore, we need to develop a cross-sectional management system in each region of the world that is convenient and not laborious or time-consuming.

We observed an age difference between the pre- and post-TB groups. The post-TB group was significantly younger than the pre-TB group. This age difference is probably due to the difference in the ability to collect medical information. We suspect that many young and enthusiastic caregivers became aware of the TB at our facility through the website and subsequently visited our institute. Nowadays, people are conscious of future life planning for ageing patients^28^. As they expect early detection and treatment for possible better outcomes^29,30^, this supports the importance of establishment of TBs.

As we showed, the post-TB group was younger than the pre-TB group, and thus, establishment of a TB may facilitate earlier detection and optimal treatment. This may lead to better epilepsy control^31^ and developmental outcomes^32^.

Although we found no significant difference between the pre- and post-TB groups in terms of prenatal diagnosis (p = 0.053), the number of patients may increase with implementation of this system and may become significant. This may also lead to early diagnosis and better outcomes in the future.

AF was diagnosed more frequently in the pre-TB group than the post-TB group. This may be due to the older age of the pre-TB cohort and the fact that AF becomes more apparent with age.

The usage rate of mTOR inhibitors was significantly higher in the post-TB group than the pre-TB group. This increase in the use of mTOR inhibitors was seen in the post-TB group not only because of the establishment of the TB but also because these drugs became available in Japan in 2012.

**Figure 1:**
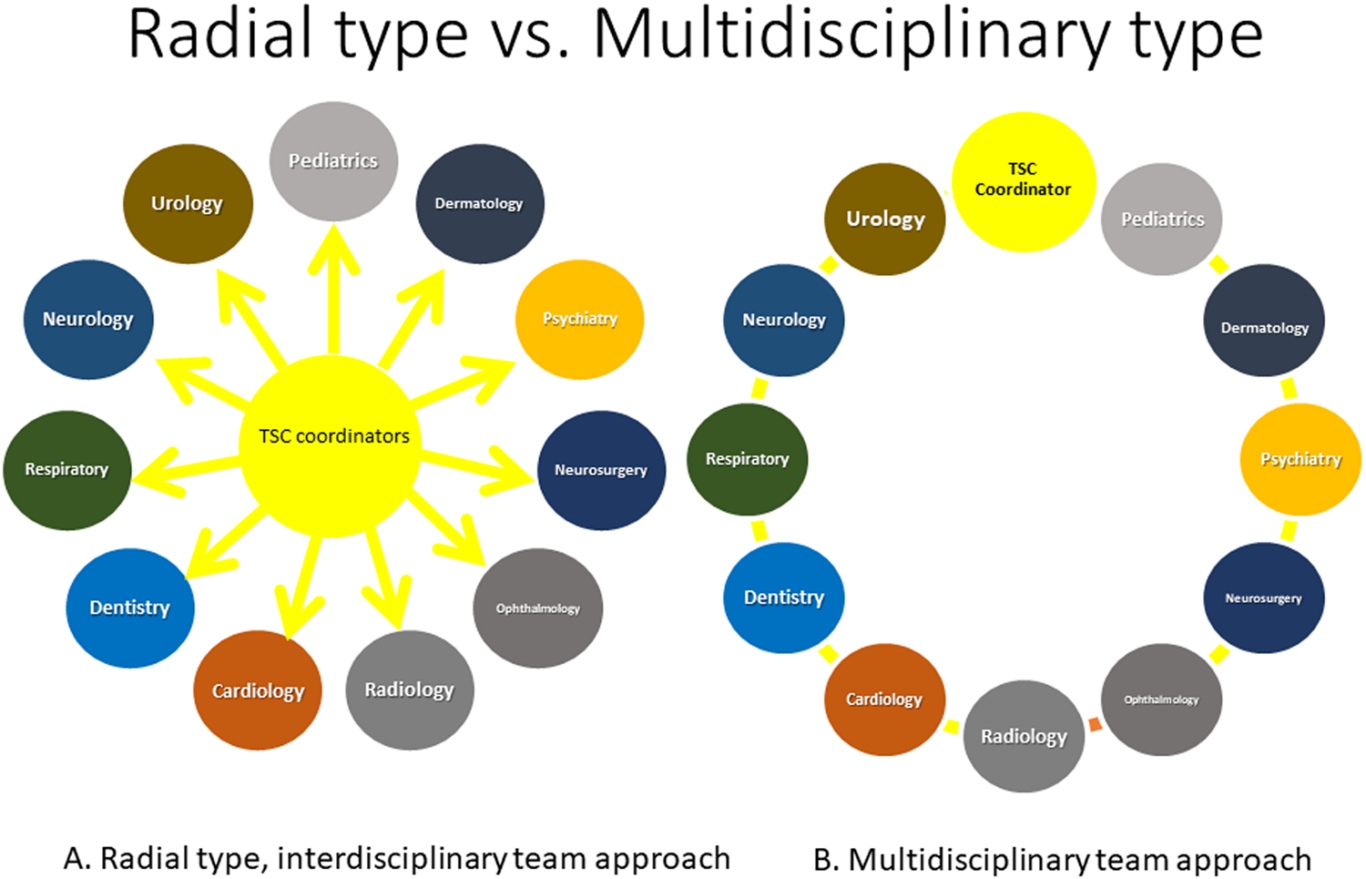
Radial type and Ring type. Coordinators are present in the axis of the departments (**A**). The coordinators are the core of the tuberous sclerosis complex (TSC) board and function like directors who bridge the relationship. In contrast, the ring type does not have a core and may not function as well in the system (**B**).

Currently, mTOR inhibitors are effective for treating SEGAs^17,33,34^, AML^35,36^, AF^37,38^, lymphangioleiomyomatosis^36,39,40^, epileptic seizures^21,41^, and others^42^, and thus, physicians’ attention to mTOR inhibitors is increasing. As the mTOR pathway may be associated with autism^43,44^, epilepsy^45^, focal cortical dysplasia^46^, and Sturge-Weber syndrome^47^, use of mTOR inhibitors may produce better outcomes in various neurological disorders. Specialists may prescribe mTOR inhibitors according to dysfunction of the organ system in which they specialize, and this may lead to confusion when specialists do not communicate with each other. This is one of the reasons for establishment of a cross-sectional TB in our facility. Once physicians share the fact that mTOR inhibitors are effective in this condition, doctors should be organically linked to each other, allowing the TB to function optimally. To enhance success of the TB, TSC coordinators, which consist of neurosurgeons, pediatric neurologists, and urologists, in our hospital serve as the axis of the system that bridges specialists. This radial relationship around the axis may be the key for optimal function of the TB (Fig. 1A). Without this axis, the TB may not function well (Fig. 1B).

As most countries do not have strong family communities such as those present in the UK or US, establishing a functioning compact TB in each medical center would be useful.

We hope that our establishment of the TB serves as a model for other institutes, and we also hope to increase the number of other successful TBs in the world.

## Methods

### Study design

Participants in this cross-sectional, observational, non-randomized study were identified via a retrospective chart review of patients treated between August 1979 and August 2017 at the Comprehensive Epilepsy Center, Seirei Hamamatsu General Hospital (Hamamatsu, Japan).

### Patients

From August 1979 to August 2017, 84 patients with TSC visited our facility. The patients’ medical records were reviewed, and patients who fulfilled the following criteria were selected: (1) met Roach’s clinical diagnostic criteria^48^; (2) had consistent reliable medical records.

We excluded patients who only had paper medical records but who did not have current electronic medical records due to inconsistent information. We also excluded patients who were diagnosed with TSC at another hospital without any other objective evidence at our hospital such as MRI, computed tomography (CT), or ultrasonography. Seventy-six patients met the criteria.

### TB

To see patients with TSC cross-sectionally and efficiently in our facility, in November 2014, we established the TB to include the Departments of Neurosurgery, Pediatric Neurology, Urology, Neurology, Dermatology, Dentistry, Respiratory, Ophthalmology, Radiology, Psychology, and Special Nurses, as well as the Medical Welfare Section and Regional Collaboration Section.

First, we established a core that consisted of three sections: neurosurgeons, pediatric neurologists, and urologists, who served as TSC coordinators (Fig. 1A). If a patient was referred from another hospital or was newly suspected in our hospital as having a diagnosis of TSC, the TSC coordinators saw the patient first and discussed the case among them. The patient was sent to see each specialist and underwent examinations and treatment according to the advice of the coordinators.

We named the period before establishment of the TB as the pre-TB era and the period after the board establishment as the post-TB era.

We reviewed age, sex, follow-up period, examinations, and treatments, and then compared these factors between the pre-TB group and post-TB group.

### Clinical information

Clinical information including age, sex, and follow-up period is shown in Table 1.

### Dermatological manifestations

Hypomelanotic macules, AF, shagreen patches, and ungual fibromas were reviewed.

### Neurological manifestations

We reviewed whether patients had epileptic seizures, how many anti-epilepsy drugs they were taking, and whether they had received other epilepsy treatments such as epilepsy surgery, VNS, or a ketogenic diet. Cortical tubers detected by MRI, CT, or ultrasonography were included in this study. We defined the intellectual function of the patients as normal if the full IQ of the Wechsler Adult Intelligence Scale III, Wechsler Intelligence Scale for Children, or developmental quotient (DQ) was ≥70, or if patients were verbal and independent in their lives. We defined moderate impairment as a full IQ ≥ 50 and <70, DQ ≥ 30 and <70, or patients who were verbal but dependent on others in their lives. Severe impairment was defined as <50 for IQ, <30 for DQ, or patients who were non-verbal and dependent on others in their lives. We defined patients with neuropsychiatric disorders as those who exhibited psychiatric events such as aggressive behaviors, saw psychiatrists, and were taking psychotropic drugs. We did not include autism spectrum disorder or psychosocial disability among the neuropsychiatric disorders in this study.

We also reviewed SEGAs, hydrocephalus, and subependymal nodules.

### Prenatal diagnosis

We reviewed the medical chart and referral letters to investigate whether the patients were prenatally diagnosed with TSC.

### Renal manifestations

Renal AML and renal cysts detected by MRI, CT, or ultrasonography were reviewed.

### Cardiac manifestations

Cardiac rhabdomyomas detected by ultrasonography and contrast-enhanced CT were reviewed.

### Pulmonary manifestations

Lymphangioleiomyomatosis and multifocal micronodular pneumocyte hyperplasia detected by CT were reviewed.

### Dental manifestations

Dental enamel pits detected by our dentists were reviewed.

### Ophthalmological manifestations

Retinal manifestations including hamartomas were reviewed.

### Statistical analysis

We statistically compared the clinical data, survey rate of each examination, occurrence of each clinical manifestation, and rates of neurological examination, nephrological examination, and mTOR inhibitor treatment between patients in the pre-TB group and the post-TB group. We used Welch’s t test, Fisher’s exact test, and Mann-Whitney’s U test as appropriate. We reported the test used in the tables. Statistical significance was set at p < 0.05. All analyses were done using JMP^®^ 10 (SAS Institute Inc., Cary, NC, USA).

### Ethical approval

Written informed consent was obtained from all patients, and procedures performed in this study were in accordance with the principles of the Declaration of Helsinki. This study was approved by the Ethical Committee of Seirei Hamamatsu General Hospital.
